# Delinquency from a Psychic Standpoint

**Published:** 1916-12

**Authors:** Jno S. Turner

**Affiliations:** Dallas, Texas


### TEXAS MEDICAL JOURNAL

Dr. F. E. Daniel, Founder. Established July, 1885

MRS. F. E. DANIEL, - Publisher and Managing Editor

Published Monthly.—Subscription, $1.00 a Year.

VOL. XXXII AUSTIN, DECEMBER, 1916 No. 6

The publisher is not responsible for views of contributors

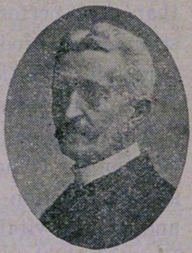

*"All of good the past hath had,
Remains to make our own time glad."*—WHITTIER.

### Original Articles.

### Delinquency From a Psychic Standpoint.

BY DR. JNO S. TURNER, DALLAS, TEXAS.

Each of the organs of the human body have distinct functions to perform, some of them having but few and comparatively simple functions, while others have numerous and very complex functions to perform in order to complete the scheme of action of the human economy. For instance, the stomach has a part of the digestive function for its duty; the liver has another part of the same process as its complement of the work. The chyle and the chyme and the blood each have very important duties to perform in order that the human machine may be kept in perfect running order. Then we have the ductless glands and the great central nervous system that has many functions which are as yet but imperfectly understood. We know that the great thyroid gland—located at the lower part of the neck, has much to do with our physical and mental growth and development, that if this gland is over developed that we have an interference with the central nervous system, that growth is retarded, that eyesight is impaired, that the heart beat is accelerated and weight is lost, and upon the whole the person is in an unstable condition. If this gland is under developed we have mental and physical lassitude, obesity, stupor, a slower than normal heart action and finally dementia. We know that there are other organs in this class, such as the pancreas, the suprarenal capsule and the ovaries, which produce a very profound impression upon the individual when their functions are impaired. We know but imperfectly just how these organs act, nor do we know the number of functions that they perform. We do know, however, that over or under stimulation produces abnormal and perverted action. The same principle of action is applicable in a marked degree to the governor of all these organs—the brain.

The brain as the central organ of control has many complex functions. It not only must control every kind of physical and secretory action of bone, muscle and fiber, including the blood and all the vital secretions, but it also must control the mental, the ethical, the moral and every other impulse or suggestion that is thrust upon it by the various special senses of the body or by the stored up or automatic action of its own organism. With these multitudinous functions of the brain in your mind, I desire that you also get fixed the natural law of development. It is a well known fact that the more often an act is repeated the more adept and certain becomes one's ability to perform the said act and the greater the development of the part involved. For instance, the feats of the acrobat, the pugilist or wrestler. The parts of the body involved in the performance of these acts become developed beyond the normal and at the expense of other parts of the body.

The brain is no exception in this particular. It is made up of numerous nerve centers all connected up by cortical and subcortical nerve fibers which we call tracts. These tracts, when applied to the thoughts, we speak of as thought currents.

These thought currents are influenced by many things, both conscious and unconscious, among which are heredity, environment, automatic action in the form of memory, etc.

The co-ordination of the various functions of the thought currents already mentioned, as well as others unmentioned, forms what we call thoughts, ideas, judgment—in fact, the mind. Therefore as these thought currents are developed in the child, so the mental and physical attitude of the child develops. It has been said that "You cannot make a silk purse out of a sow's ear," and very truly so. But by proper psychic training you can prevent the silk purse from degenerating and great improvement can be made on the "sow's ear."

Three things largely influence the psychic life of the child,—namely, heredity, environment and health. These three things being determining factors as to whether the child is to develop into a well rounded, normal individual or into a dependent or delinquent.

It has been said that the potent period of potentiality in the life of a child is during the first five years; to be sure, these years are more prolific of development that will bear upon its future life than any other similar period.

At this time in the child's life the mind is as a white sheet of paper or a film that has as yet to be exposed to the light. The mind is in a plastic state and susceptible to any influence that happens to be directed its way.

We note the influence of heredity upon the normal child in its features, its favor to the father or the mother, in the complexion, in its temperament, in its likes and dislikes, in its talents, musical, artistic or otherwise, and in many things we see in the child the counterpart of its forbears. Nature has ordained that "like begets like," hence the continuity of natural law. Mausley says: "There is a destiny made for a man by his ancestors, and no one can elude, were he able to attempt it, the tyranny of his organization."

While this is true in the abstract, it is also true that by properly directing the thought currents during the plastic period will in a measure overcome many defects of mind. As has been suggested, development and growth comes with repeated and systematic use of a part. In the case of the blacksmith or hammer thrower, the arm and its muscles are systematically stimulated, the blood vessels are dilated by development, more blood is thereby carried to that part and an increased general growth occurs. After this development comes automatic action or the action of "second nature," as it is sometimes called; by that we mean that it comes natural for the performer to do certain feats that at first was quite difficult of performance and required the concentration of every power possessed by body and mind for the accomplishment even in an imperfect manner.

This same physical law of development and automatic action is applicable to psychical things. The thought currents can be stimulated along certain lines of action at the expense of the thought currents relating to other lines, until the selected routine of thoughts will be the line of least resistance, building up the capillaries and arterioles of that part of the brain out of normal proportion. We see the musician in his first attempt to play upon the instrument; the musical center of the brain as well as the physical center controlling his fingers are as yet untutored and it is with the greatest difficulty that he makes even the most imperfect note. After years of practice and the acquirement of automatic action of the brain centers and the "tactus eruditus,"

or educated touch, we see him again, developing the most wonderful music, and apparently with perfect ease and as though it were natural, even doing so while he converses animatedly upon a topic foreign to his music.

We have seen the child of ignorant and superstitious parentage —the Indian if you please—after he had been given the advantage of an education, beginning even much later than the plastic age of five years and after the uncivilized instincts had been well developed. The thought currents of his earlier training, cultivation and development being almost obliterated by the training of another set of thought currents which were developed at the expense, so to speak, of the first mentioned. While the hereditary influences were not destroyed, they were so weakened by the counter influences that he was able to allow judgment and finally will power to direct the course of his future life, whereas without this beneficent training of the dormant thought currents he would have possessed and practiced all the savage inheritance of his race.

So with the child of bad inheritance, if it can be gotten early—the earlier the better—and can be directed along lines of proper thought during the plastic, or so-called formative period—there is reason for much hope. But taken late in life, after the thought habits have been formed and the thought current development has been thoroughly established and there is less hope of regeneration or reform.

The same rule will apply to the child of good parentage or heredity if placed early in a bad or delinquent environment, if the suggestions that are made to its plastic mind are of the kind that stimulates thoughts of profanity, dishonesty, unchastity, delinquency, etc., it is pretty sure to develop along that line instead of the hereditary line. It is believed by most observers, however, that the delinquent of good inheritance has the better ultimate chance of reform when taken late in life, than has the delinquent of bad heredity.

After years of training of the thought currents that stimulate thinking along delinquent lines, it is really a very difficult matter for an individual to think consecutively and sustain that line of thought for any great length of time, upon any subject except it be in line with delinquency. At this point suggestion plays a prominent role. I have no doubt but that many of you have many times seen delinquents who seemed thoroughly penitent and who promised upon honor that they would reform, only to fall as soon as out from under the influence of the suggestion of your strong will power. It would not be fair to these people to say that they *all* were imposters and were not sincere in their promise to do better or to reform, for many of them no doubt were sincere, and while under the influence of your strong and wholesome suggestion had the strongest kind of desire to reform, and when they promised to do better fully believed that they would do better. But after leaving that wholesome influence and being thrown upon their own thought resources, the introspection that comes with that responsibility carried them in thought along the line of least resistance—the delinquent line—and not having the proper counter thought current suggestion to assist them in their good intentions, it was easy to again fall.

It is difficult to know the potentiality of an individual, because we do not know how much of the good inheritance there may be lying dormant in the undeveloped and dwarfed brain centers. We cannot know how much influence we have had over whatever of good that is lurking in those centers nor how strong a counter current we may have aroused, hence we should not give up finally a delinquent until all effort at reform has proven futile.

Three prime factors enter psychically into the making of a delinquent, namely, the time element; alcohol, and ill health. In the first we recognize the natural law in the force of development. The physiological axiom that the last centers to be developed in life are the first to go when mental change comes about, is equally applicable to delinquency. We believe that the first line of delinquents or those who sprang immediately from good parentage are easier to reform and can be reformed at later ages, as has already been suggested, than those who have two or more generations in delinquency behind them. After that length of time delinquency has become almost a racial characteristic and it will take proper training and surroundings for one or more generations to eradicate those characteristics. We see the white bear, the white fox and the light hair and blue eyes in the extreme cold climates; we see the black bear and the black races in the hot climates; we see the gypsy in his roving disposition all being racial characteristics, and it takes change of environment and some generations in time to change these characteristics; likewise with other racial or family characteristics and likewise with the delinquent who comes of a long line of delinquents. Another potent factor is alcohol. When a people or an individual, from heredity or otherwise, permits the moral standard of life to become dwarfed, the physical side of life will become accentuated. The normal flow of vitality in the individual must be directed into some channel under self-control, else the physical or the animal will predominate. When that occurs the appetite becomes the ruling passion, as is the case in the purely animal kingdom. The day that appetite becomes enthroned is coronation day for King Alcohol, and the future progress and development of that individual or of that people is in line with such orgies as follows its excessive use. When the individual is given over to alcohol, the heart's action is increased, the circular fibers of the blood-vessels are dilated and since the capillary circulation of the surface of the body and the cortex of the brain is the largest system, the warm blood that should be resident in the internal organs of the body for the proper functionation of the same is attracted to the surface and to the cerebrum or large brain, thus lowering the physical vitality of the individual and increasing abnormally the excitability of the centers in control of the thought currents. When this condition—intoxication—occurs the high tension placed upon the delicate structure of the thought currents and the overflow of blood into the minute capillaries of the brain tend to unbalance or unstabilize that delicate organ, producing short circuits, as it were, in the thought currents so that judgment, the product of automatic action from long storing of experience in the thought centers in the form of memory, is held in abeyance, and without this judgment or inhibitory power the individual acts upon impulse and in an erratic manner. The moral element of the brain centers being the last to be developed, is the first to suffer when this brain change takes place, and then the animal passions are in full possession of the individual.

In this state of intoxication, when animal passion is supreme, many conceptions occur, and often when both parents are in the same condition, and as the inexorable law of "like begets like" is in full effect, what are we to expect of offspring from this type of parentage? Certainly delinquency.

By way of illustration of the effect of heredity and alcoholism I will give you the statistics on two well known and often quoted families—namely the Jurke family and the Kallikak family, both were families traced from degenerate mothers.

In the Frau Jurke family there were traced 834 descendants, with the following findings: Illegitimates, 106; beggars, 142; on the charity of the State, 64; lewd females, 181; criminals of all grades, 169. This family had cost the commonwealth $1,250,000.

The Kallikak family is but little better. In this family a man had two wives, a legitimate wife, who was of sound mind, and an illegitimate wife of the high grade imbecile class. There was traced from the legitimate sound-minded mother 496 descendants, with no defectives. From the illegitimate unsound-minded mother there was traced 480 descendants, with the following history: Feeble-minded, 143; undetermined, 293; known to be normal, only 44.

We now come to the largest class of delinquents and to the largest cause of delinquency. I refer to psychical ill health; due to either prenatal or acquired causes. During an alcoholic debauch, when judgment is inhibited, or after all moral sense is gone, or perhaps in a legitimate manner by a diseased mother or father a child is conceived to be born a degenerate, and certainly doomed to become a delinquent. The end result of this prenatal transmission is represented by the large class of imbeciles of various grades, idiots, epileptics and consumptives, as well as by many physical monstrosities and degenerates. The acquired causes are represented by those children who contract various diseases by the use of the common drinking cup, the common towel and by careless association with the unclean. Many children become disease infected by this character of association while at school. These diseases, covering the whole field of transmissible disorders, but the most direful and far-reaching of them all, are the venereal diseases.

Other acquired diseases which alter the psychic potentiality of the child and tend to delinquency by way of lowered mentality, are hyperthyroidia, enlarged tonsils, adenoids, chorea, tuberculosis, defective vision, chlorosis in young girls, inanition and improper housing among the poor, and dementia precox from overwork and under-nutrition.

A report issued by the United States Bureau of Education estimates that of the 20,000,000 school children of this country, at least 75 per cent need attention to some defect, which tends ultimately to lower vitality and to retard both physical and mental progress. This report particularizes by stating that:

"From 1½ to 2 per cent, or 400,000, of these have organic heart disease.

"Probably 5 per cent, or 1,000,000, at least, have now or have had tuberculosis of the lungs.

"About 5 per cent, or 1,000,000, have spinal curvature, flat foot or some other moderate deformity serious enough to interfere to some degree with health.

"Over 5 per cent, or 1,000,000, have defective hearing.

"About 25 per cent, or 5,000,000, have defective vision.

"About 25 per cent, or 5,000,000, are suffering from malnutrition, in many cases due, in part, at least, to one or more of the other defects enumerated.

"Over 30 per cent, or 6,000,000, have enlarged tonsils, adenoids or cervical glands which need attention.

"Over 50 per cent, or 10,000,000 (in some schools as high as 89 per cent) have defective teeth, which are interfering with health.

"Several millions of the children possess, each, two or more of the handicapping defects."

Many of these children show little or no evidence of mental defect until they are about to enter the age of adolescence, at which time the vitality is somewhat taxed and an arrest of psychic development occurs from which they never recover.

When the brain centers are in this subnormal state, attention is difficult to arrest, and, therefore, concentration is imperfect; for this reason, in many cases, there is more or less psychic blindness. The physical eye observes and reflects images upon the lens and the retina in a faithful manner and the nerve of special sight sense takes up the stimulus and conveys it to the psychic sight center, but since that center, together with most of the other psychic centers, has been obtunded by lack of proper development, it either does not take any notice or at best but imperfect attention is given the stimulus, and in this way no psychic impression is made upon the film of the mind and memory is unable to perform its function of storing up these impressions for future use, hence, when the time for action on the matter arrives and the subject is again presented to the psychic centers there is little or no memory of the former impression, and, therefore, there is no previously formed judgment on the matter, and the individual must act without the discretion that comes of judgment and solely from the standpoint of premature impulse. The same principle applies to psychic hearing and to the responses psychically from impressions made by the other special senses; and since about 65 per cent of our information and education is acquired by the process of observation and the storing on the mental film through memory, and since the defective person is incapacitated, in that particular, we can begin to understand why they are unable to become self-reliant and are more or less dependent. Being dependent in the same ratio that they are more or less psychically defective. We see many of these cases who seem to be quite accomplished in petty thievery, trickery, malingery and in the art of petty crimes and vices, and we wonder why they are so apt along these lines yet so deficient along moral lines. We also see in our every-day business relation men and women who seemed to be normal, even especially endowed along certain lines, but who seem to be utterly devoid of common sense with relation to other matters of life. These people while not considered defectives (in fact, many of them are brilliant) are yet of the poorly balanced type, psychically speaking, and have developed in "a lop-sided" manner, so to speak. A. limited number of their faculties and centers having been over stimulated, cultivated or educated at the expense of the whole. We see much of this in the devotees of the arts and sciences, among actors and highly trained specialists in many subjects.

Self-preservation being the first law of nature, of necessity the delinquents have had to practice such mental devices and powers with which they were endowed in order to secure a living, and since they are unable to contest in the same fields with those psychically normal, they must of necessity select the line of least resistance and proceed along a route which is comparatively but little frequented, and lacks keen competition.

By sheer force of circumstances, then, they are driven into the field of petty vice and crime, and since centering all their mental powers on the one idea, crime and vice, they further dwarf what little ability they might have had for other things, and the same is compensated for by a rather remarkable upbuilding of the centers devoted to their modus operandi. Their cupidity should be a lesson and act as an encouragement to the social worker, for it is proof positive that the imbecile, whether he be of the high, intermediate or low grade is possessed of some potentiality and if properly reached, directed and re-educated is susceptible of much improvement. It has been said that the brain cells suffer for their supremacy over other tissue, by being unable to reproduce themselves when once destroyed.

While that is true and we cannot, therefore, hope to restore an imbecile to the station of a normal person, regardless of the diligence of our efforts, yet it is also true that in the case of high grade and many intermediate and some low grade imbeciles, much can be accomplished by a diligent and patient process of re-education.

---

Mrs. A. R. Lewis, the wife of Dr. A. R. Lewis, of Ryan, Oklahoma, passed to her reward November 28. She was much loved by those who knew her, and her death will be mourned by a host of friends.

